# Correlates of health and financial literacy in older adults without dementia

**DOI:** 10.1186/1471-2318-12-30

**Published:** 2012-06-12

**Authors:** Jarred S Bennett, Patricia A Boyle, Bryan D James, David A Bennett

**Affiliations:** 1 Rush Alzheimer’s Disease Center, Rush University Medical Center, Chicago, USA; 2 Department of Behavioral Sciences, Rush University Medical Center, Chicago, USA; 3 Department of Internal Medicine, Rush University Medical Center, Chicago, USA; 4 Department of Neurological Sciences, Rush University Medical Center, Chicago, USA

**Keywords:** Health literacy, Financial literacy, Older adults, Dementia

## Abstract

**Background:**

Recent research has begun to recognize the important influence of literacy levels and how they affect health and wellbeing, especially in older adults. Our study focuses on health and financial literacy, two domains of literacy which previous research has suggested may be significantly related to health and wellbeing. Our study examines the relation of health and financial literacy with health promoting behaviors and health status among community-based older persons.

**Methods:**

We conducted a cross-sectional study using data from the Rush Memory and Aging Project, a community-based cohort study of aging in northeastern Illinois. The study consisted of 556 older persons without dementia, each determined by a clinical evaluation. Health and financial literacy were measured using a series of questions designed to assess the ability to understand and process health and financial information, concepts, and numeracy; the two scores were averaged to yield a total literacy score. Health promoting behaviors, including engagement in cognitive, physical, and social activities, were assessed using self report measures. Indicators of heath status, including cognition (global cognition and five specific cognitive abilities), functional status (basic and instrumental activities of daily living, mobility disability), and mental health (depressive symptoms, loneliness) were assessed.

**Results:**

In a series of regression models adjusted for age, sex, and education, higher total literacy scores were associated with more frequent participation in health promoting behaviors, including cognitive, physical and social activities (all p values <0.05). Higher total literacy scores were associated with higher cognitive function, less disability, and better mental health (all p values < 0.05). Literacy remained associated with health promoting behaviors and health status in fully adjusted models that also controlled for income and the number of chronic medical conditions. Most of the findings were similar for health and financial literacy except that health literacy was more strongly associated with health promoting behaviors whereas financial literacy was more strongly associated with mental health.

**Conclusions:**

Health and financial literacy are associated with more frequent engagement in health promoting behaviors and better health status in older persons without dementia.

## Background

Historically, literacy referred to the three technical skills of reading, writing and calculating. Over the past half century, a plural notion of literacy has evolved such that literacy is now considered a multi-dimensional construct involving a host of skills essential for optimal functioning in society and the achievement of one’s personal goals. The United Nations Educational, Scientific and Cultural Organization (UNESCO) defines literacy as “the ability to identify, understand, interpret, create, communicate and compute, using printed and written materials associated with varying contexts. Literacy involves a continuum of learning in enabling individuals to achieve their goals, to develop their knowledge and potential, and to participate fully in their community and wider society.” [1]. In this context, many Americans display low levels of literacy. About 90 million adults scored poorly on the 2003 National Assessment of Adult Literacy [2]. Low literacy is a particularly significant problem among persons over age 65, the majority of whom score below basic competency levels [2]. This represents a particular challenge since late life is a time at which some of life’s most significant and influential health and financial decisions must be made (e.g., health insurance plan selection, complex medical options, retirement savings, inter-generational transfers). Literacy, therefore, encompasses a wide range of competencies, including the domains of health and financial literacy, both of which have important implications for the health and well-being of older persons [3-15], the fastest growing segment of the population in developed countries [16,17]. We define health literacy as the ability to access, understand, and utilize health information and concepts in ways that promote good health outcomes, and we define financial literacy as the ability to access, understand, and utilize financial information and concepts in ways that promote good financial outcomes, as previously reported [18] and consistent with prior work [19-21]. Our definition of health literacy is consistent with the definition used by the Institute of Medicine which is “The degree to which individuals have the capacity to obtain, process, and understand basic health information and services needed to make appropriate health decisions.” [20]

Recent studies of older persons have shown that low health literacy is associated with poorer self-rated health and increased all cause mortality [3-6]. Persons with low health literacy also have higher health care costs and are less efficient in their use of health care services [7,8]. However, there is limited data on the relation of health literacy to health promoting behaviors such as social, physical, and cognitive activity. Studies have reported associations with physical activity [6] and use of preventive services [9], but other health promoting behaviors have not been examined.

Further, whereas studies of older persons have shown that financial literacy is important for financial planning in old age [22], very little data from older persons are available regarding the relation of financial literacy with health promoting behaviors and measures of health status. However, evidence from younger persons suggests that financial literacy may also have important health consequences, especially with regard to mental health. For example, the British Household Panel Survey (BHPS) found that higher financial capability was associated with better self reported mental health and better self reported physical health [23].

Most prior studies of the relation of literacy to health in older persons used screening tests of cognition or functional abilities or medical record review to exclude persons with moderate to severe dementia [6]. However, they did not perform clinical evaluations to exclude persons with dementia, and specifically mild dementia. Thus, it remains unclear whether mild dementia accounts for some of the associations of literacy with health outcomes. In this study, we examined the correlates of literacy among more than 550 community based persons from the Memory and Aging Project, all of whom were clinically evaluated and determined to be without dementia. We collected data on health and financial literacy and created a total literacy score based on the average of these two domains. Then, in a series of analyses adjusted for age, sex, and education, we examined the relation between total literacy and health promoting behaviors, including cognitive, physical, and social activity, all of which have been associated with adverse health outcomes. We then examined the relation between total literacy and several indicators of health status, including cognition, functional status, and mental health. Additional analyses considered potential confounders. We then examined these associations separately for health and financial literacy.

## Methods

### Participants

Participants were 556 older adults from the Rush Memory and Aging Project, an ongoing longitudinal clinical-pathological study of common chronic conditions of aging. Study participants were recruited from about 40 subsidized housing facilities and retirement communities in the Chicago metropolitan area. Participation in the study includes risk factor assessment and annual clinical evaluations. The literacy assessments were added to the Memory and Aging Project evaluation in 2008. The study was approved by the Institutional Review Board of Rush University Medical Center, and informed consent was obtained from each participant following a detailed presentation of the risks and benefits associated with study participation. At the time of these analyses, 600 participants had completed the baseline clinical evaluation and the decision making evaluation. Subjects with dementia were excluded from analyses. Each subject underwent a uniform clinical evaluation that included a medical history, neurological examination, and cognitive function testing, as previously described [24]. The diagnosis of dementia was made by a clinician experienced in the assessment of older persons and followed the National Institute of Neurologic and Communicative Disorders and Stroke and the AD and Related Disorders Association criteria [25], which require a history of cognitive decline and evidence of impairment in at least two cognitive domains. Of the 600 participants who had completed the health literacy and financial literacy assessments, 31 who met the criteria for dementia were excluded, and 556 (97.7%) of the remaining 569 had complete data on literacy. Participants with mild cognitive impairment (MCI) were those who were judged by the clinician to have cognitive impairment but of insufficient severity to warrant a diagnosis of dementia; persons with MCI were not excluded to ensure the sample was not composed of “super-agers.”

### Health and financial literacy assessment

Health and financial literacy were measured using a series of questions designed to assess the ability to understand and process health and financial information, concepts, and numeracy, as previously described [18]. Participants were given nine questions on health literacy, including questions that measure understanding of Medicare and Medicare Part D, instructions for taking medications, common causes of death in older persons, and a question requiring participants to interpret the risk of taking a drug with prespecified outcomes. There were 23 questions on financial literacy, many of which were adapted from the Health and Retirement Study [26]. Questions assessed numeracy by asking participants to perform simple mathematical calculations and to answer questions that reflect the ability to understand financial concepts such as compound interest or inflation rates. Other questions assessed knowledge of financial terms and institutions such as FDIC, stocks, and mutual funds. All questions were either multiple choice or true/false. All items were scored as correct or incorrect. Since the health and financial literacy tests contained a different number of items, the two subscores were expressed as the percentage correct out of total items (from 0–1). The total literacy score was the average of these two percentages. Total literacy had adequate internal consistency with a Cronbach’s coefficient alpha of 0.77.

### Cognitive activity assessment

Frequency of participation in cognitive activities was measured with a previously established scale [27]. Persons rated their current frequency of participation in nine cognitively stimulating activities (e.g., reading a newspaper, playing games like chess or checkers) on a 5-point scale with 5 indicating participation in the activity every day or about every day and 1 indicating participation once a year or less. The scale focuses on common activities in which seeking or processing information is vital and which has minimal physical or social requirements. The scores from each of the nine items were averaged to yield a composite measure of cognitive activity that has been shown to have adequate short term temporal stability and positive association with cognitive function and level of educational attainment [27,28], and association with risk of AD and mild cognitive impairment (MCI) and cognitive decline [29].

### Physical activity assessment

Physical activity at baseline was measured with questions adapted from the 1985 Health Interview Survey [30]. Persons were asked if they had participated in each of five activities (e.g., walking for exercise, yard work) during the past 2 weeks, and if so, the number of occasions and mean time per occasion. The durations of each activity were summed and expressed as a number of hours per week of physical activity, as described elsewhere [31]. This measure has been associated with a variety of adverse health outcomes including decline in motor function and incident disability [31,32].

### Social activity assessment

We used a previously established composite measure of late-life social activity in these analyses [33]. For frequency of participation in social activities, respondents were asked to rate how often they participated in six common social activities (e.g., visiting friends o relatives, participating in groups) on a 5-point scale, ranging from almost every day to once a year or less [34,35]. Item responses were summed and averaged to yield a composite measure of social activity frequency. This measure has been associated with a variety of adverse health outcomes including decline in motor function, incident disability, and cognitive decline [34-37].

### Cognition

Cognitive function was tested with 21 performance tests as previously described [24]. The Mini-Mental State Examination (MMSE) [38] was used for descriptive purposes but not in analyses. One test was only used for diagnostic classification. Cognitive tests assessed episodic memory (seven tests), semantic memory (three tests), working memory (three tests), perceptual speed (four tests), and visuospatial ability (two tests) [28]. A composite measure of global cognition, based on all 19 tests was formed by converting each subscore on component tests to z-scores, using the baseline mean and SD, and computing the average.

### Functional status

Physical function was assessed with three self report measures. The self report measures of disability evaluated the degree of difficulty experienced in six basic activities of daily living (e.g. bathing, dressing), eight instrumental activities of daily living (e.g. housekeeping, shopping), as well as three measures of mobility disability (e.g. walking up and down flight of stairs). Participants rated their ability to perform the above activities by answering whether they needed help, did not need help, or were unable to perform the activity at all.

### Mental health

There were two self report measures of mental health. We assessed depressive symptoms with a 10-item form [39] of the CES-D Scale. Items (e.g., “I felt sad”) were read to participants, who then indicated whether they had felt that way much of the time during the past week. The number of symptoms endorsed has been associated with morbidity and mortality in previous research in older persons [40]. Loneliness was assessed with a modified version of the de Jong-Gierveld Loneliness Scale [41]. The original version of this scale has been shown to be internally consistent [42]. The modified version has been previously reported and shown to be associated with adverse health outcomes including risk of AD [42].

### Other covariates

Demographic information was collected at baseline. Age was self-reported and was based on date of birth and date of the health and financial literacy assessment. Sex and education (years of schooling) were also self-reported. Chronic medical conditions were the sum of self-reported medical condition items (hypertension, diabetes, heart disease, cancer, thyroid disease, and head injury with loss of consciousness) as previously reported [43]. Income was measured using the show card methodology, as previously described [24]. Self-reported annual income at baseline was ranked according to 10 possible categories: 1: $0–$4999, 2: $5000–$9999, 3: $10,000–$14,999, 4: $15,000–$19,999, 5: $20,000–$24,999, 6: $25,000–$29,999, 7: $30,000–$34,999, 8: $35,000–$49,999, 9: $50,000–$74,999, 10: >$75,000.

### Statistical analysis

We first examined bivariate associations of literacy with demographics and all covariates via Pearson and Spearman correlations as appropriate. We then used linear regression models to examine the cross-sectional associations between total literacy and health promoting behaviors and health status. We first examined the associations using models adjusted for age, sex, and education. We then ran models that further adjusted for income and chronic medical conditions. In additional analyses, we repeated the above analyses separately for health and financial literacy. Analyses were carried out in SAS version 9.2 [44].

## Results

### Descriptive properties of the study cohort

Descriptive characteristics are presented in Table 1. The mean age was 83, mean education was 15 years and 76% were women. The mean score on the Mini-Mental State Examination (MMSE) [38] was 28.2 (SD = 1.8). Twenty eight percent of the participants had self reported annual income lower than $25 K, 37% had income between $25 K and $50 K, and 35% had income over $50 K. On average, participants had one or zero medical conditions (mean = 0.75, SD = 0.9). The mean total literacy score was 66% (SD = 15%); the mean health literacy score was 61% (SD = 19%) and the mean financial literacy score was 72% (SD = 16%). Higher health literacy, financial literacy, and total literacy were correlated with younger age, more education, and males tended to have higher literacy than females. The correlation between health literacy and financial literacy was 0.46 (p < 0.001).

**Table 1:** Characteristics of the study cohort

**Characteristics**	**N**	**Mean**	**SD**
**Demographic**			
Age	556	82.720	7.430
Sex	425	76.44%	
Education	556	15.243	3.004
**Total literacy**	556	.663	.152
Financial literacy	556	.718	.164
Health literacy	556	.608	.191
**Health promoting behaviors**			
Cognitive activity	555	3.156	.683
Physical activity	556	3.354	3.470
Social activity	556	2.589	.588
**Cognition**			
Global cognition	556	.229	.543
Episodic memory	554	.339	.724
Perceptual speed	548	.105	.820
Working memory	556	.134	.700
Visuospatial ability	543	.233	.735
Semantic memory	548	.251	.604
**Functional status**			
Basic activities of daily living	556	.403	1.041
Instrumental activities of daily living	556	1.491	1.917
Self reported mobility disability	553	.980	1.152
**Mental health**			
Depressive symptoms	556	1.061	1.674
Loneliness	556	2.198	.623
**Covariates**			
Baseline income	488	6.990	2.432
Medical conditions	556	.766	.896

### Literacy and health promoting behaviors

We first examined the relation of literacy with cognitive activity. In a model adjusting for age, sex, and education, higher literacy was associated with higher cognitive activity score (Table 2-Core Model, estimate for cognitive activity in core model). Based on the estimated association, a person with a total literacy score of 0.86 (90^th^ percentile) had a cognitive activity score of 0.64 standard deviations higher than a person with a total literacy score of 0.41 (10^th^ percentile). The results were similar in fully adjusted models that also controlled for chronic medical conditions and income (Table 2-estimate for cognitive activity in the fully adjusted model). In additional analyses, the associations of health literacy and financial literacy with cognitive activity were also similar in both the core and fully adjusted models (Table 2).

**Table 2:** Association of health, financial, and total literacy with Health Promoting Behaviors and Health Status in core and fully adjusted models*

**Characteristic**	**Total literacy**		**Health literacy**		**Financial literacy**	
	**Beta coefficient (SE,*p*value)**		**Beta coefficient (SE,*p*value)**		**Beta coefficient (SE,*p*value)**	
	**Core**	**Fully adjusted**	**Core**	**Fully adjusted**	**Core**	**Fully adjusted**
**Health promoting behaviors**						
Cognitive activity	.108 (.021, <.001)	.078 (.023, .001)	.059 (.016, <.001)	.041 (.017, .016)	.100 (.020, <.001)	.075 (.022, .001)
Physical activity	.249 (.111, .025)	.225 (.126, .074)	.185 (.083, .026)	.188 (.092, .041)	.159 (.105, .129)	.090 (.121, .459)
Social activity	.056 (.018, .002)	.049 (.020, .014)	.038 (.014, .005)	.033 (.015, .025)	.043 (.017, .012)	.034 (.019, .076)
**Cognition**						
Global cognition	.200 (.013, <.001)	.188 (.015, <.001)	.124 (.011, <.001)	.112 (.011, <.001)	.162 (.013, <.001)	.154 (.015, <.001)
Episodic memory	.214 (.021, <.001)	.190 (.022, <.001)	.138 (.016, <.001)	.119 (.016, <.001)	.166 (.020, <.001)	.144 (.022, <.001)
Perceptual speed	.222 (.023, <.001)	.214 (.024, <.001)	.141 (.017, <.001)	.132 (.018, <.001)	.175 (.022, <.001)	.168 (.024, <.001)
Working memory	.187 (.021, <.001)	.176 (.023, <.001)	.100 (.016, <.001)	.090 (.017, <.001)	.176 (.019, <.001)	.169 (.022, <.001)
Visuospatial ability	.134 (.022, <.001)	.140 (.024, <.001)	.083 (.016, <.001)	.082 (.018, <.001)	.109 (.021, <.001)	.118 (.024, <.001)
Semantic memory	.195 (.016, <.001)	.184 (.018, <.001)	.116 (.012, <.001)	.107 (.013, <.001)	.162 (.016, <.001)	.155 (.018, <.001)
**Functional status**						
Basic ADL	−.076 (.033, .022)	−.026 (.032, .419)	−.063 (.025, .011)	−.041 (.023, .080)	−.033 (.031, .286)	.023 (.031, .451)
Instrumental ADL	−.334 (.055, <.001)	−.259 (.056, <.001)	−.222 (.042, <.001)	−.174 (.041, <.001)	−.252 (.053, <.001)	−.178 (.054, .001)
Self reported mobility disability	−.144 (.035, <.001)	−.142 (.039, <.001)	−.101 (.026, <.001)	−.102 (.029, <.001)	−.102 (.033, .001)	−.086 (.038, .023)
**Mental health**						
Depression	−.155 (.053, .004)	−.097 (.058, .095)	−.061 (.040, .130)	−.040 (.043, .350)	−.178 (.050, <.001)	−.111 (.056, .048)
Loneliness	−.036 (.019, .057)	−.042 (.021, .047)	−.015 (.014, .284)	−.019 (.015, .225)	−.044 (.018, .014)	−.045 (.020, .026)

*Core models adjusted for age, sex, and education. Fully adjusted models also control for income and chronic medical conditions.

Abbreviations: ADL, Activities of daily living.

We next examined the relation of literacy with physical activity. Higher literacy was associated with more frequent participation in physical activity (Table 2, Core Model, estimate for physical activity in the core model); that is, a person with the 90^th^ percentile total literacy score had a physical activity score about 0.29 standard deviations higher than a person with the 10^th^ percentile total literacy score. However, in the fully adjusted model, the association was similar but was no longer significant. In additional analyses, higher health literacy was associated with more frequent physical activity in the fully adjusted model, but financial literacy was not associated with physical activity.

Finally, in the core model, literacy was associated with social activity such that a person with 90^th^ percentile total literacy score had a social activity score about 0.40 standard deviations higher than a person with a 10^th^ percentile total literacy score based on the core model estimate (Table 2). Results were similar in fully adjusted models. The associations of health literacy and financial literacy with social activity were also similar in core and fully adjusted models, although the latter was no longer significant at α = 0.05 for financial literacy.

### Literacy and health status

We first examined the relation of literacy with global cognition and five specific cognitive abilities. Literacy was associated with cognition such that in the core analysis of global cognition, a person with the 90^th^ percentile total literacy score had a global cognition score about 1.51 standard deviations higher than a person with the 10^th^ percentile total literacy score (Table 2). Results were similar in fully adjusted model. The associations with health and financial literacy were also similar in both the core models and the fully adjusted models. In addition, total literacy, and health literacy and financial literacy, were all also significantly associated with five specific cognitive domains in both the core models and the fully adjusted models.

We next examined the relation of literacy to functional status. Literacy was associated with all three measures of functional status (Table 2). However, in the fully adjusted models, total literacy was only associated with instrumental activities of daily living and self-report mobility disability. The results were similar for health literacy.

In the final set of analyses, we examined the relation of literacy to mental health. In the core analysis, literacy was related to both depressive symptoms and loneliness (Table 2). However, in the fully adjusted models, depressive symptoms failed to reach statistical significance. Financial literacy was associated with both indices of mental health whereas health literacy was not.

## Discussion

We examined the relation of literacy with health promoting behaviors including cognitive, physical, and social activities, and indicators of health status including cognition, functional status, and mental health in a community-based cohort study of over 550 older individuals clinically evaluated and determined to be free of dementia. We found that total literacy was associated with participation in cognitive and social activities. It was also associated with better cognition, less impairment in instrumental activities of daily living, less mobility disability, and less loneliness. In additional analyses of the two domains of literacy separately, similar associations were frequently present for both health and financial literacy. However, in general, health literacy was more strongly associated with health promoting behaviors, whereas financial literacy was more strongly associated with indices of mental health. Overall, the results suggest that literacy is associated with participation in health promoting behaviors and indicators of health status. Our findings raise the possibility that strategies to improve health and financial literacy may have the potential to improve public health, even in advanced age.

Most prior work on the relation of literacy with health has focused on the domain of health literacy. One prior study showed an association between health literacy and physical activity [6]. Prior studies also have reported an association between health literacy and indices of health status, including cognitive function [12] and functional status, including ability to perform instrumental activities of daily living [9,13]. Health literacy has also been related to indices of mental health, with one study showing an association between health literacy and depressive symptoms [4], while another did not [45].

While there has been less focus on the relation of financial literacy with health promoting behaviors and health status, data from several studies have shown direct and indirect associations between financial literacy and health. For example, cognition, a component of health status, has been related to indices of financial literacy [14]. One study also reported an association between numeracy and self-reported health [12]. A recent study found that the presence of real estate owned properties (2005–2009) in a neighborhood, an indicator of foreclosure rates, was associated with increases in medical visits for a variety of mental health conditions such as anxiety among persons 18–64 [46]. In addition, the Greek minister for health reported an increase in suicide rates of about 40% in the first months of 2011 that were thought to be attributable to unemployment [47].

The present study builds on prior literature in three important ways. First, we excluded persons with dementia based on a detailed clinical evaluation. It is well accepted that dementia is associated with impaired medical and financial competence [48,49]. An important limitation of prior work is that screening methods for dementia consisted of excluding persons with a MMSE score below 18 [10], impairment in basic activities of daily living [6], or exclusion based on medical record review [7]. These approaches may identify moderate to severe forms of dementia but will not eliminate many persons with mild dementia. Our findings restricted to persons without dementia suggest that the previously reported associations with health and financial literacy are not due to the confounding effects of dementia. Second, we extend previous research by showing that health literacy is associated with participation in cognitive and social activities, in addition to physical activity. Cognitive and social activities, like physical activity, are health promoting behaviors associated with a wide range of adverse health outcomes, including disability, dementia and death [29,31,32,34,36,37] and are targets of intervention studies actively underway in the elderly. Third, we found that financial literacy, a domain not well studied in older persons in relation to health, was also associated with cognitive activity and indices of health status including cognition, functional status, and mental health. In fact, the findings with mental health were more robust for financial literacy than for health literacy. As there were many more items in the financial literacy scale, it is possible that these findings were the result of a psychometrically more stable measure rather than being specific to financial literacy. By contrast, it may be that financial literacy has a relatively selective effect on mental health. Financial literacy may make individuals more confident and secure about their future, particularly in later life when there are limited opportunities to overcome financial mistakes. It has been suggested that lack of financial literacy may contribute to financial stress and lead to poor mental health [50], and our findings support and extend this idea in the elderly. There is also data suggesting that higher financial capability and higher financial literacy are associated with better psychological wellbeing [10,11]. In this way, financial literacy may affect indices of health indirectly through its impact on mental health. Some studies report that mental health is associated with a variety of adverse health outcomes in older adults [35,36,40,42,43]. Furthermore, one study found that communities with higher foreclosure rates, which may be indicative of lower financial literacy, experience higher hospital admission rates [46]. Thus, our data are consistent with the hypothesis that financial literacy impacts health status through an effect on mental health. However, further prospective studies will be needed to clarify these associations.

The finding that literacy is associated with health promoting behaviors and indices of health status is novel and has important public health policy implications. About 90 million adults scored poorly on the 2003 National Assessment of Adult Literacy, and the majority of persons over age 65 scored below basic competency literacy levels [2]. In addition, persons over age 65 suffer a disproportionate burden of disease and are faced with some of life’s most significant financial decisions. It certainly is the case that greater financial literacy is associated with better financial outcomes, and financial status is related to access to and utilization of healthcare services. Financial literacy most likely also is related one’s knowledge of the important role of lifestyle factors as determinants of health outcomes, and persons with greater financial literacy (and financial status) may be more likely to have the opportunity and resources needed to engage in health promoting behaviors (i.e., access to and resources to pay for fruits and vegetables and gym memberships). Further, the Foresight Program in the United Kingdom Government Office for Science recently issued a report on mental capital and wellbeing [51] which found strong links between mental health problems and debt. One of the recommendations was better financial management training to help break the cycle between debt and mental illness. Our findings extend previous research and raise the possibility that strategies to improve health and financial literacy have the potential to improve public health even in advanced age. That being said, there is increasing evidence that the seeds of age-related chronic diseases are sown in early and mid life [52]. Thus, efforts to improve health and financial literacy should not only target older persons, but also persons of younger ages [15].

There are several strengths to this study. We used a structured multistep process to diagnose dementia, and those with dementia were excluded from analyses to reduce confounding. Our participants were older than participants in most prior studies resulting in a cohort at greater risk for adverse health outcomes allowing examination of literacy and health in later life. We assessed a range of novel health promoting behaviors. We also measured cognitive function using a detailed cognitive battery that yields a global measure and measures of five important domains of cognition. We also assessed a broader array of indices of functional status and mental health. Finally, we assessed associations with financial literacy, a topic not well studied in relation to health among older persons. The study also has important limitations. MAP participants are a volunteer cohort, and findings based on their health and literacy levels may not be representative of the general population of older adults. Thus, studies in more representative populations are needed. In addition, the study is cross-sectional and we cannot determine the direction of causality. For example, it is possible that literacy is related to or a function of cognitive decline even among persons without dementia. Longitudinal studies of the relation of literacy to health will be important to determine the causal directions and will inform on the likelihood that interventions to improve literacy will result in improved public health. Finally, we did not use common measures of health literacy such as the Rapid Estimate of Adult Literacy in Medicine (REALM), which measures the ability of adults to pronounce and recognize medical terms [53], or the Test of Functional Health Literacy in Adults (TOFHLA), which measures the ability of adults to perform basic reading tasks needed to function in the health care environment, such as reading prescription bottle labels, understanding appointment slips, and completing health insurance forms [54]. This makes it difficult to compare our findings to prior work in the literature and it is possible that our results would have differed had we used one of these other measures. While the REALM and TOFHLA have strengths, we believe our measure also has strengths in terms of identifying the ability to understand and process health concepts specifically relevant to older persons; assessing literacy in this manner may provide novel information about the knowledge older persons need to make decisions that promote good health outcomes consistent with the 2004 Institute of Medicine’s definition of Health literacy [20].

## Conclusions

Health and financial literacy are associated with more frequent engagement in health promoting behaviors and better health status in older persons without dementia. The data raise the possibility that improvements in literacy in older adults may lead to better physical and mental health, potentially resulting in more efficient use of health and financial services, increased self-sufficiency, and lower health care costs. More research is needed to determine the causal pathways linking literacy with health promoting behaviors and health status in order to develop interventions and strategies that will most effectively promote physical and mental health in older adults.

## Competing interests

None of the authors have a relevant competing interest.

## Authors’ contributions

JSB drafted the manuscript. PAB helped manage the data and edit the manuscript. BDJ helped performed the statistical analysis and helped edit the manuscript. DAB helped to coordinate the study, manage the data, and edit the manuscript. All authors read and approved the final manuscript.

## Pre-publication history

The pre-publication history for this paper can be accessed here:

http://www.biomedcentral.com/1471-2318/12/30/prepub
